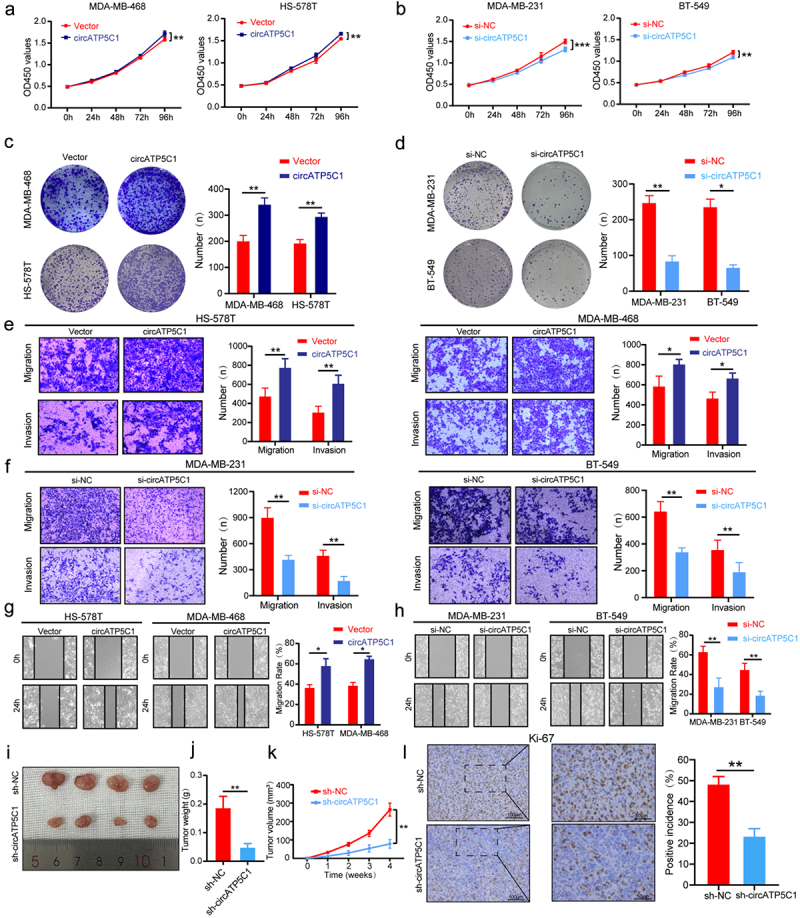# Correction

**DOI:** 10.1080/15384047.2025.2491914

**Published:** 2025-04-11

**Article title**: CircATP5C1 promotes triple-negative breast cancer progression by binding IGF2BP2 to modulate CSF-1 secretion

**Authors**: Liu, H., Wang, H., Gao, W., Yuan, Y., Tang, T., Sang, M., Liu, F., & Geng, C.

**Journal**: *Cancer Biology & Therapy*

**DOI**: https://doi.org/10.1080/15384047.2025.2479926

The author has identified an error in Figure 3. The figure has been updated, and the author requests that the figure be replaced with the revised version provided below, as it more accurately reflects the original intentions

**Figure 3:**